# Composite Materials Design: Biomineralization Proteins and the Guided Assembly and Organization of Biomineral Nanoparticles

**DOI:** 10.3390/ma12040581

**Published:** 2019-02-15

**Authors:** John Spencer Evans

**Affiliations:** Laboratory for Chemical Physics, Center for Skeletal and Craniofacial Biology, New York University, 345 E. 24th Street, New York, NY 10010, USA; jse1@nyu.edu; Tel.: +1-347-753-1955

**Keywords:** biomineralization, mesocrystal, nanoparticles, particle attachment, proteomics, nucleation, biocomposites, hydrogels

## Abstract

There has been much discussion of the role of proteins in the calcium carbonate biomineralization process, particularly with regard to nucleation, amorphous stabilization/transformation, and polymorph selection. However, there has been little if any discussion of the potential role that proteins might play in another important process: the guided assembly and organization of mineral nanoparticles into higher-ordered structures such as mesocrystals. This review discusses particle attachment theory and recent evidence of mineral-associated proteins forming hydrogels that assemble and organize mineral clusters into crystalline phase. From this discussion we postulate a mechanism by which biomineralization protein hydrogel aggregation assists in mineral nanoparticle assembly and organization within calcium carbonate skeletal elements and discuss potentials ways for harnessing this process in materials design.

## 1. Introduction

In Nature, numerous oceanic organisms build protective and functional skeletal elements using calcium carbonate [1,2]. These skeletal elements often have interesting material properties such as fracture resistance, resistance to crack propagation, puncture resistance, and even optical properties. In many instances these skeletal elements are biocomposites consisting of one or more calcium carbonate polymorphs that contain organic macromolecules [1,2]. These organic components are synthesized by the organisms and introduced into the mineralization space at the appropriate time. With the genomic sequencing of calcium carbonate-based organisms [3,4,5,6,7], we now know that numerous protein families [8,9,10,11,12] comprise this organic component and are affiliated with the mineral matrix. The question is, what are these proteins doing there?

Well, the answer is not so straightforward. For some time, scientists have pondered the role or function of biomineral-associated matrix proteins, individually and collectively, in the calcium carbonate mineralization process [1,2]. There has been some discussion of protein mediation of major biomineralization mechanisms, such as the nucleation of amorphous calcium carbonate (ACC) [13,14,15,16,17,18,19] and the transformation of ACC into a polymorphic crystalline form [13,14,15,20,21,22,23]. In addition, certainly, one cannot logically argue against proteins playing some role in these processes. However, recently scientists have discovered another important process that occurs in skeletal element formation, and that is the assembly of ACC or crystal nanoparticles into larger biogenic mesocrystals [24,25,26], such as the calcitic spicule of the embryonic sea urchin [21,27,28] and the aragonitic tablets of the mollusk shell nacre layer [21,29,30]. In each tissue, there is evidence that the mesocrystals are comprised of numerous nanoparticle subunits that have a limited range of particle sizes [21,27,28,29,30]. These phenomena have led scientists to develop a revised picture of the biomineralization process: crystals form from a non-classical route involving some form of liquid-liquid phase separation [22] or guided particle assembly [31]. This has been discussed in a recent theory known as crystallization by particle attachment (CPA) [31]. Here, a crystal forms as a result of nanoparticle aggregation into an amorphous mineral phase, which then transforms via Ostwald ripening into a crystalline phase [22,31].

The question is, does the guided assembly of nanoparticles proceed without assistance [24,25,26], or, is there a need for assistance, and if so, do proteins assist in this process? This review will present a hypothetical mechanism regarding protein-directed guided particle assembly using recent data obtained for the sea urchin spicule and the mollusk shell nacre skeletal elements. The argument is as follows: the mineralization process requires guided particle assembly in both skeletal elements and proteins are adept at assembly [32,33,34]. A potential mechanism would involve the incorporation of nanoparticles within protein matrix aggregates, which then assemble into higher-ordered structures, thus positioning the nanoparticles into a mesocrystal. This process is completely consistent with known protein functionalities of macromolecular interaction and assembly [32,33,34] and would partly explain why proteins were incorporated into the biomineralization process over evolutionary time.

## 2. Biogenic Mesocrystals and Their Nanoparticle Components

### 2.1. The Sea Urchin Spicule

**Figure 1:**
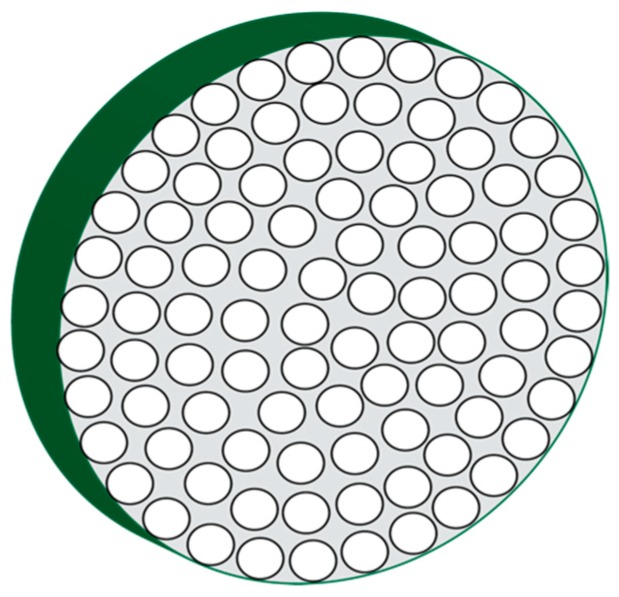
Cartoon representation of a high-magnification transverse cross-section through a developing sea urchin spicule, revealing radial, coaxially arranged calcium carbonate mineral nanoparticles. Adapted from references [21,27].

The mineralized spicule develops into a single crystal that is actually a mesocrystal consisting of a concentric arrangement of mineral nanoparticles (Figure 1) [21,23,27]. The ellipsoidal to circular coaxial cross-section of the spicule reflects the fact the spicule forms from a tube-like collection of cells known as the syncitium [35,36,37], which deposits macromolecules and mineral-containing vesicles and then this tubular cell collection gradually withdraws in a radial direction as the matrix is formed [21,27,35,36,37]. This leads to the formation of radially oriented spherical mineral nanoparticles. What is interesting about these mineral nanoparticles is that they possess a coating, the nature of which is not completely known, but is speculated to be proteinaceous in origin [27], and, is known to have intracrystalline proteins residing within the mineral phase [16,19,35,36,37].

### 2.2. The Mollusk Shell Nacre Layer

**Figure 2:**
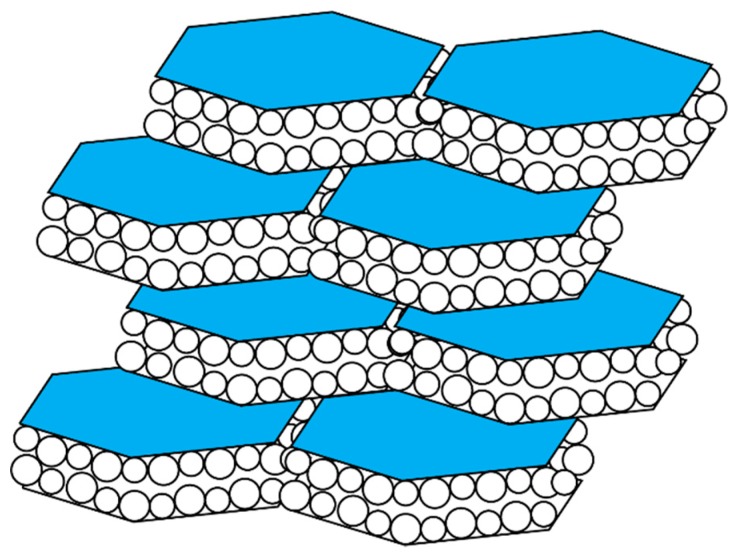
Cartoon representation of a high magnification oblique cross-section through a mollusk shell nacre layer, consisting of “brick and mortar”-arranged hexagonal aragonite tablets that are comprised of spherical mineral nanoparticles. Adapted from references [38,39,40,41,42].

The aragonite nacre tablets of the mollusk shell are hexagonal in appearance and are organized into a “brick and mortar” arrangement (Figure 2) [23,38,39,40,41,42]. These tablets possess an external organic coating that is comprised of the beta-chitin polysaccharides, nacre-specific proteins, and a silk-fibroin protein [23,38,39,40,41,42]. Like the spicule, each of the tablets consists of a closely packed arrangement of spherical mineral nanoparticles that fits within the hexagonal confines of the tablets and, the tablets are known to possess intracrystalline proteins [23,38,39,40,41,42].

## 3. Hydrogels and Biomineralization

**Figure 3:**
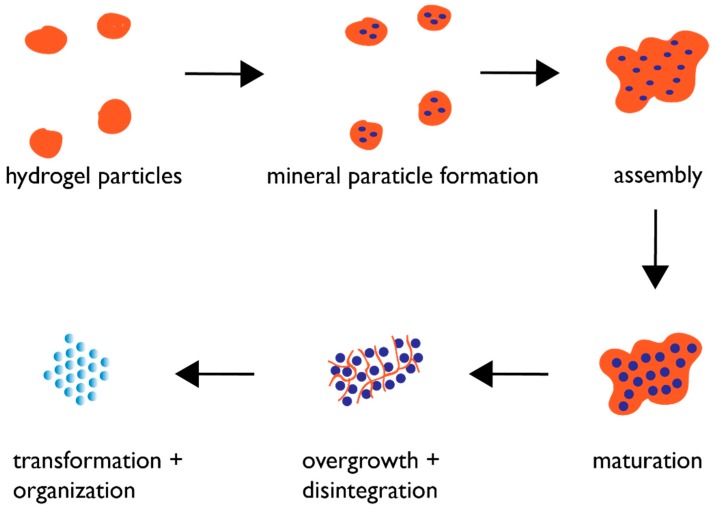
Proposed protein hydrogel pathway for biogenic mesocrystal formation. Blue circles denote mineral nanoparticles. Maturation stage refers to the growth of the ACC nanoparticles to permissible dimensions within the hydrogel matrix. The overgrowth and disintegration stages refer to the continued mineral growth and simultaneous protease degradation of the hydrogel matrix, resulting in matrix capture within the mineral nanoparticles as intracrystalline organic nanoinclusions.

The formation of crystals or nanoparticles in bulk solution represents a disorder-to-order entropic transformation with thermodynamically important barriers to be overcome [21,22,23,24,25,26,27,28,29,30,31]. If we examine the ultrastructure of the resulting mineral phases in Figure 2 and Figure 3, it is evident that the mineral phase is highly ordered, much more so than one would expect to happen in bulk solution without the addition of additives. The nucleation process is chaotic, with wide distributions of mineral particle dimensions forming over time and no real evidence of organized or guided assembly. If this process were to occur solely on its own, then it would be nearly impossible for the organized structures of Figure 2 and Figure 3 to occur over any timescale. Therefore, that means that the nucleation process must have a guidance system imposed upon it in order to achieve hierarchal organization.

And this is where biomineralization proteins come into play. The very first “Ah Ha!” moment came with the discovery that the mollusk shell nacre layer actually consisted of a hydrated gel-like phase [43,44,45]. This phase was attributed to the major protein fraction, the silk-fibroin protein family, that was known to inhabit the nacre layer and the polysaccharide, beta-chitin, also a resident of this same layer [44,45]. It was also recognized that several nacre protein families were affiliated with this hydrated silk–chitin phase as well, and that these nacre proteins exerted influence on the nucleation process within the nacre layer [10,20,44,45]. This is really an important revelation: that a biomineralization process takes place within a hydrogel environment.

However, why is a hydrogel so significant? A hydrogel possesses three critical properties that would be advantageous to have vis a vis mineral formation and crystal growth [46,47,48,49,50,51]. First, there is volumetric confinement, which has been shown to be important for amorphous phase stabilization [18,20] and crystal growth [20,52,53]. In the case of a hydrogel, the internal pores suffice as “limited volume reaction vessels”, where nucleation would be permissible, but amorphous solid formation would be restricted in dimension by the surrounding gel matrix [20,46,47,48,49,50,51]. Second, the gel matrix would affect ion and water diffusion, such that the movement of each will impact nucleation kinetics and hydration/dehydration processes, respectively [20,46,47,48,49,50,51]. In turn, these events can affect ACC formation, stabilization, and transformation. Third, hydrogels form as a result of assembly of polymer chains [20,46,47,48,49,50,51], and thus there is the potential for hydrogel-associated mineral nanoparticles to be co-assembled as the hydrogel forms. Thus, the presence of a hydrogel matrix within a mineralization environment is impactful, and we speculate that many skeletal elements arise from hydrogel- or sol-gel controlled biomineralization processes.

## 4. Biomineral-Associated Protein Families Are “Smart” Hydrogelators

What evidence is there for hydrogel-forming (hydrogelation) proteins within skeletal elements? Recent experimental studies have shown that seven shell-specific [43,54,55,56,57,58,59,60,61,62,63,64,65,66,67,68,69,70,71,72] and two spicule-specific proteins [73,74,75,76,77] form hydrogels in the presence and absence of Ca(II) at pH 8.0, the relevant pH of the calcium carbonate mineralization process [43,54,55,56,57,58,59,60,61,62,63,64,65,66,67,68,69,70,71,72,73,74,75,76,77]. These hydrogel particles exhibit porosities and irregular morphologies, and these features, along with dimension and organization, change in response to variables such as pH, ions, and ion concentrations [59]. By definition, these are “smart” or responsive hydrogelators [46,48,49], and their functions in the mineral matrix would be expected to change as the mineralization process evolves (i.e., as Ca(II) and carbonate/bicarbonate concentrations and matrix pH change over time).

There are several ways in which a “smart” hydrogel can impact the mineralization process. One of these is to influence the hydration or dehydration of ionic clusters or ACC clusters, and, influence the timeline for nucleation [78,79,80,81,82]. Recent experiments indicate that some biomineralization protein hydrogels can bind and release water [57,60,75], which could impact the stability and transformation of nearby ACC clusters [78,79,80,81,82]. Potentiometric experiments with both nacre and spicule matrix proteins confirm that this is the case: hydrogels affect the solubility and stability of ACC [43,56,57,58,60,61,62,63,65,76,77]. Furthermore, these hydrogels can also affect the movement or availability of ionic species [57,75], leading to prolonged nucleation times [43,56,57,58,60,61,62,63,65,76,77]. These same features would also manifest themselves when protein hydrogels deposit onto existing crystals, which would affect surface hydration and ion availability, leading to changes in surface morphologies or crystal growth directions [58,66,67,73,76,77].

Why do these biomineralization proteins act as hydrogelators? From previous studies [13,14,15,43,56,57,58,60,61,62,63,65,76,77,83], we have learned that it comes down to the primary sequence and three specific features therein: (1) intrinsic disorder, or sequences which are thermodynamically unstable and do not form folded structures. These sequences destabilize the protein and create an extended structure which facilitates a sol-gel or gel-like state; (2) amyloid-like aggregation-prone sequences, which are cross-beta strands, that promote self-aggregation and protein-protein aggregation; (3) a globular or folded domain that enables protein–protein interaction. Thus, with these sequence elements present in these proteins it is clear that matrix or hydrogel formation would occur easily in solution.

## 5. Evidence for Protein-Driven Particle Assembly and Organization

Given that protein hydrogels can induce mineral particle organization on crystal surfaces, the next logical step would be for these same hydrogels to perform particle organization at earlier stages of crystal growth. The first demonstration of this came with STEM flow-cell studies of a nacre protein, AP7 that formed and assembled mineral particles into ring-like structures in solution [65]. These events are evidence for a directed assembly process.

The second demonstration of protein guided particle assembly and organization was documented recently for two sea urchin spicule matrix proteins, SM30B/C and SM50 [73]. Here, using MicroCT (µCT) imaging [56], each protein was individually observed to produce a stratification of mineral deposits over time, whereas the control scenario did not generate significant stratification. However, when both spicule matrix proteins were present together in the same assay, these proteins formed a hydrogel matrix that not only limited mineral particle formation but also assembled and organized the mineral particles into discreet domains or clusters. This phenomenon was not observed in control scenarios, indicating that the two spicule matrix proteins were responsible for the observed assembly and organization of the mineral particles [65]. Thus, both nacre and spicule-specific proteins are capable of directed particle assembly and most likely give rise to the organized nanoparticle-based mesocrystal that we observe in both skeletal systems (Figure 1 and Figure 2).

## 6. Proposed Mechanism of Protein-Directed Nanoparticle Assembly into a Crystal Format

Based upon the findings of recent studies [43,54,55,56,57,58,59,60,61,62,63,64,65,66,67,68,69,70,71,72,73,74,75,76,77], we believe that biogenic crystal formation in the skeletal elements of the mollusk shell and sea urchin spicule obey the following hypothetical mechanism (Figure 3). As the protein assembly process generates hydrogel particles within a supersaturated environment, mineral ACC nanoparticles begin to nucleate within the pore regions of these hydrogel particles [62,63,65,68]. This ACC phase is stabilized by the hydrogel against crystalline transformation at this point; presumably, this occurs via the hydrogel’s ability to regulate water availability [57,75]. The hydrogel particles then begin to assemble into larger complexes, which has been documented to occur in the presence of Ca(II) ions [43,55,56,57,58,59,60,61,62,63,64,65,66,67,68,69,70,71,72,73,74,75,76,77]. This assembly process now brings the ACC nanoparticles in closer proximity to one another, creating organized mineralized clusters. Over time, the hydrogel matrix is overwhelmed by mineral growth; portions of the hydrogel matrix become incorporated into the forming mineral phase as intracrystalline inclusions [55,58,63,64,66,67], whereas other portions are cleaved by matrix proteases [3,4,5,12,84] and no longer associate with the hydrogel, thereby reducing the ability of the hydrogel to stabilize the ACC clusters. The result is that the ACC clusters, lacking stabilization from the protein hydrogel, are susceptible to changes in hydration and can now expand in dimension and transform into the appropriate polymorphic crystalline form via Ostwald ripening [22,31] but still retain their spherical morphologies that we noted in Figure 2 and Figure 3.

## 7. The Future: Where Do We Go from Here?

If we assume that “smart” hydrogel matrices are the key to mineral particle formation and assembly, then the next stage of biomineralization research will clearly be one of further categorization. Studies could be expanded beyond calcium carbonates to include other biomineralizing systems such as calcium phosphates, silicates, magnetite, and so on, to determine if protein assembly/hydrogelation guide mineral particle assembly in those systems as well. In situ identification of protein gels or matrices that possess mineral nanoparticles would be a prime objective for verifying the hypothesis, not only in calcium carbonates but in other biomineralizing systems as well. This would involve temporal monitoring of protein expression and the association of proteins with the assembly of nanoparticles. Next, the materials and engineering fields would be interested in using this process to create synthetic inorganic assemblies and mesocrystals for technological applications. Thus, there would be a need to understand protein sequence requirements for hydrogelation and assembly and translate this into a polymer-based system that would support various inorganic assembly schemes under laboratory conditions specific for non-biogenic inorganic solids. Further investigation of hydrogelation would also be a goal, where porosities, ion and solvent exchange rates, and assembly kinetics and dimensions would be measured, then tuned for a specific nanoparticle/material system. We challenge the biomineralization and materials communities to seek out these possibilities, such that the biomineralization process can finally be understood and translated into a workable polymer-based scheme for technology and industry.
